# Comparative cataract—lens opacification across species

**DOI:** 10.1038/s41433-025-03874-3

**Published:** 2025-06-19

**Authors:** David Williams

**Affiliations:** Queen’s Veterinary School Hospital, Department of Veterinary Medicine, University of Cambridge, Madingley Rd, Cambridge, CB3 0ES UK

**Keywords:** Microscopy, Lens diseases

## Abstract

Around 12 million people globally are estimated to be blind with age-related cataract and numerous studies have sought to evaluate the causes of this lens opacification, many of these complicated by the difficulty of determining the impacts of varying aetiological factors from daylight through diet, diabetes and many more. While various experimental investigations on laboratory animals have sought to unpick these influences on lens transparency to date none has sought to evaluate age-related cataract across different animal species kept as companion animals. Here we look at a number of studies on companion dogs, cats and horses to determine the effects of daylight, diet, diabetes, dehydration, drugs, genetics (DNA to continue the D alliteration) and trauma (Damage again as a final D).

## Introduction

**Fig. 1:**
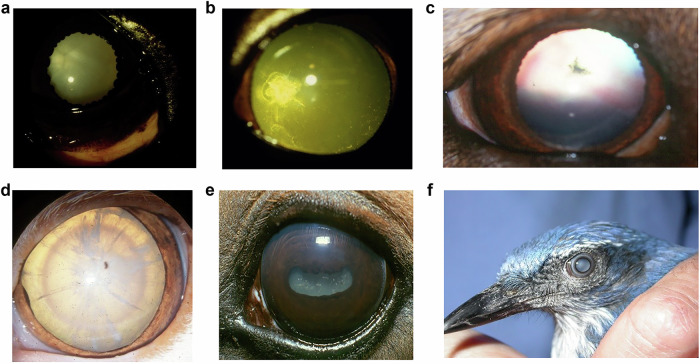
Cataracts in different companion animal species. **a** Diabetic cataract in a 12 year old springer spaniel. **b** Inherited posterior polar subcapsular cataract in a 6 year old Labrador retriever. **c** Steroid-induced cataract in a 7 year old crossbred on oral prednisolone for 2 years. **d** Diabetic cataract in a 13 year old domestic short-haired cat. **e** Mature cataract in a 22 year old thoroughbred horse. **f** Post-traumatic cataract in a 4 year old American scrub jay.

I’m not sure what I find more remarkable. That evolution could produce a transparent structure suspended in the middle of the eye perfectly engineered to focus light on the retina, or that this structure manages to stay transparent in a large proportion of individuals for the majority of their lives. And that given the fact that light shines through the lens for all the time we are awake potentially exposing the proteins in the lens to the risk of photo-oxidation that majorly contributes to ageing of other structures such as exposed skin [1]. Indeed the cells and the proteins within them in the rest of the body continually renew themselves. From an ophthalmic perspective corneal epithelial cells reduplicate every few days [2, 3] moving from their stem cell population at the limbus to the corneal centre [4]. Given that they are continually exposed to the environment one might expect that perpetual regeneration. But even in the optic nerve, hidden far from the environment, while the ganglion cell axons may be there for life apparently unchanged, the proteins of which they are constructed are in continual flux [5]. Yet the crystallin proteins in the middle of the lens are the same ones that were formed in utero. The collagens so regularly arranged in the cornea gradually change over time but those crystallin proteins in the equally transparent lens have no opportunity to be removed and replaced should they be damaged. How remarkable then that they last for the entire life of the individual, 10-15 years for a dog, 70–100 for a human maybe more than 200 years in bowhead whales [6, 7]. And for the majority of that lifespan they stay unchanged giving a transparent lens. But this is not always the case. Cataract accounts for nearly half the cases of blindness in low-income countries and 5% in high-income countries [8], so evaluating the causes of lens opacification and ways of preventing or slowing it is critical. Surgery to correct lens opacification is readily available in developed countries but much more difficult to provide in equatorial countries where the prevalence of cataract is much higher. In 1988 at the Cambridge Ophthalmological Symposium Professor Hugh Taylor spoke on the epidemiology of cataract, giving his 5 Ds of cataract aetiology [9]. In Taylor’s list Daylight came first given the molecular origin of age-related lens opacification through photo-oxidation. Diet has an influence through the influence of dietary anti-oxidants. Dehydration could have an influence though the areas where severe dehydration through diarrhoea is prevalent also have high UV levels and dietary compromise. Diabetes is certainly linked with an increased risk of cataract. Therapeutic drugs, particularly steroids are potent causes of cataract in human patients. But as Taylor admitted ‘the largest group by far is ‘Don’t Know’ and this is the category that keeps lens researchers in business.’ While in the 25+ years since that paper much evidence suggestive for those five Ds in human cataract has accrued, in this paper I’d like to add information from spontaneous cataract formation in several animal species from dogs through cats and horses to birds and reptiles (Fig. 1), and to add a further two Ds to the list.

## Evaluating cataract prevalence

**Fig. 2:**
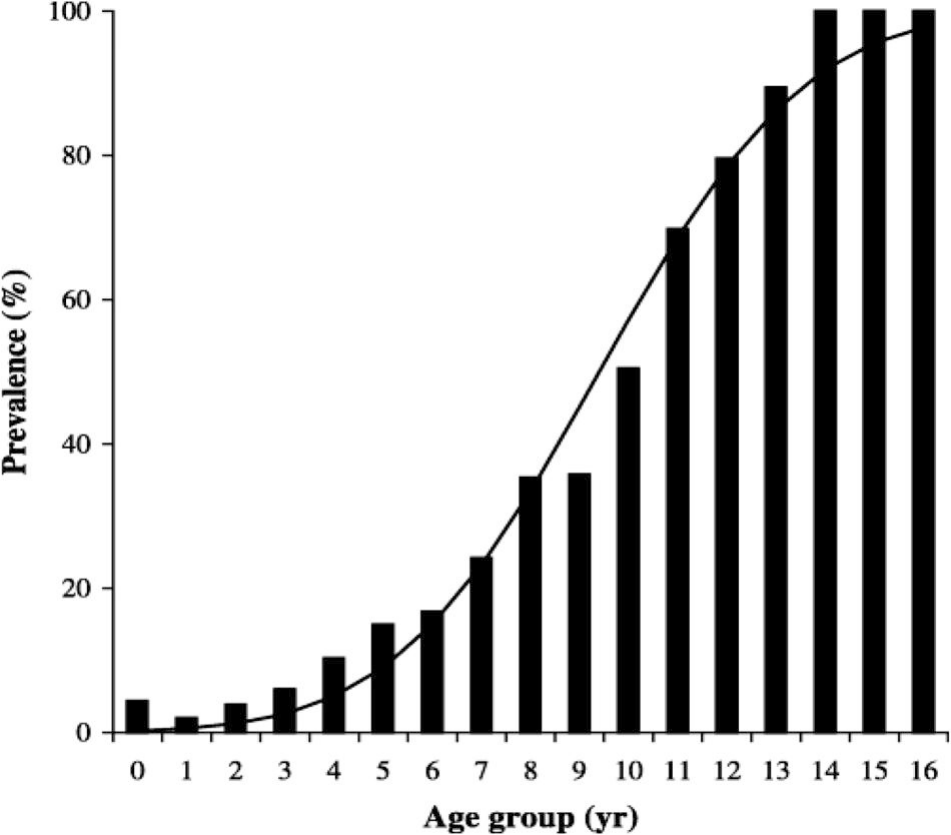
Graph of cataract prevalence by age in all 2000 dogs in Williams et al. [42].

**Fig. 3:**
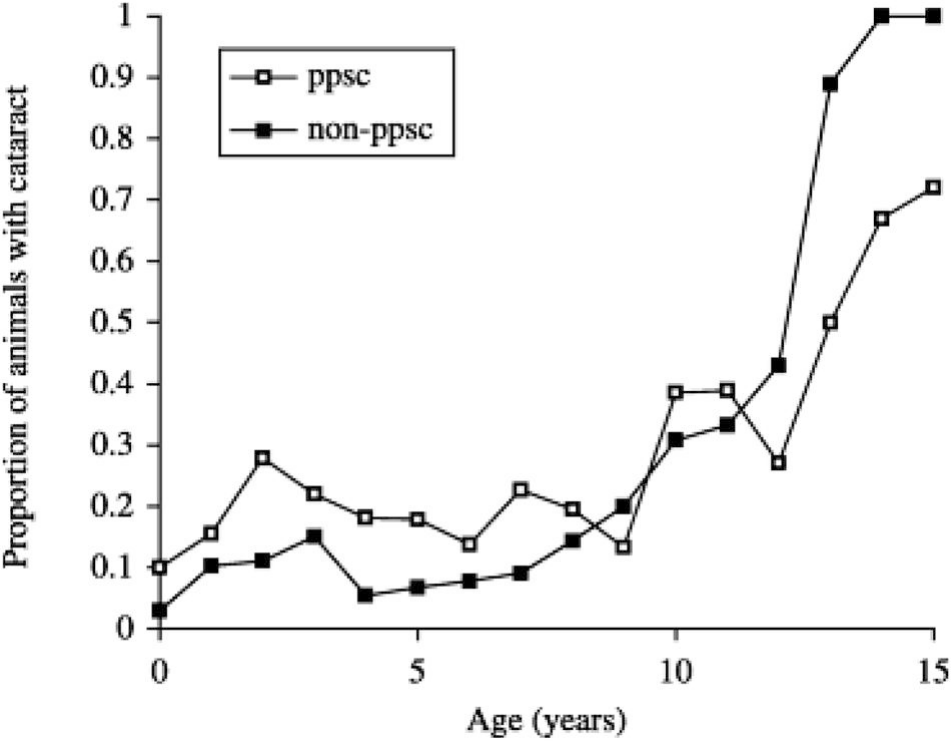
Proportion of Labrador Retrievers in each age group with posterior polar subcapsular (ppsc) cataract and other lens opacities (non-ppsc).

In the studies documented below the prevalence of cataract in each group of animals was estimated by determining prevalence of lens opacity in each year group and then determining the age at which 50% of the population showed lens opacity, henceforth termed the C_50_ value [10]. In the canine eye for instance ophthalmic examination was undertaken in 2000 companion dogs to give the graph shown in Fig. 2. The mean C_50_ value in this canine population was 9.4 ± 3.3 years. As we will see below different dog breeds with different longevities showed different C_50_ values. This technique does not however, differentiate varying cataract types nor the degree of opacity, hence it is a very rough-and-ready estimate, but one we have found helpful in determining aetiopathogenesis of age-related cataract. It does also assume that the cataracts noted are all age-relayed which may of course not necessarily be the case. Analysis of cataract prevalence in the Labrador Retriever for instance is complicated by the existence of inherited posterior polar subcapsular cataract in a sizeable percentage of the population. Other lens opacities in the breed show a C_50_ of 11.4 ± 2.2 but the graph of prevalence by age of the posterior polar subcapsular cataract (Fig. 3) shows a very different curve from that of Fig. 2, with an increased early prevalence together with a rising late prevalence. For such data a single C_50_ is not considered an appropriate measure. For the data we will consider below however, comparing cataract prevalence under different conditions of light exposure, dietary intake, dehydration, diabetes and drug use C_50_ is a valuable estimate of the prevalence of lens opacification in a population.

## Daylight

Taylor’s first D was daylight, quite rightly as incident light, particularly at the ultraviolet end of the spectrum, is a significant cause of age-related cataract. We know from laboratory studies the effects that ultraviolet light has on photo-oxidation of proteins and lipids in the lens. Epidemiological evidence can be more difficult to analyse since effects other than merely irradiation will have their effect and differ between different populations.

If we could find two genetically equivalent populations having the same nutritional input but with markedly different ultraviolet irradiances that would be highly valuable in determining the effect of daylight on cataract formation. But where might we find such populations? People in equatorial regions are likely to have very different dietary intake of antioxidants for instance, complicating the determination of the effect of light on the lens. Studies of human populations such as comparing Chesapeake Bay watermen and those assessed in the Framlingham Eye Study [11] are complicated by having different assessment systems and studying different select populations. Brilliant and colleagues, working in Nepal, as might be hoped from the name of the senior researcher, investigated sunlight exposure at high altitude, and suggested a ratio of 2.6 between Nepalese working in sunlight for 12 hours compared with those with 7-9 hours exposure [12]. There were potential problems with this study, since people working at high altitudes naturally took preventative measures to protect themselves from excessive sunlight exposure and assessing quite how much time each individual spent in direct exposure to the sun was difficult. Taylor investigated cataract in Chesapeake Bay watermen, a group with high UV exposure [13] and again found a correlation between cortical cataract prevalence and ultraviolet exposure but comparing with cataracts in miners was difficult because the miners spent their leisure time in the sun while the watermen spend more in the shade. Perhaps studying animal populations might be easier?

Quarter horses are kept in very similar environments in the UK and in Texas but for the level of UV irradiance to which they are exposed. Meteorological data for Dallas, Texas and Brentwood, Essex during the relevant periods were obtained from the United States National Weather Service Forecast Office and the Meteorological Office, Exeter. The intensity of sunlight at a given latitude equals the value at the equator (around 1370 J/m/s) multiplied by the cosine of the relevant latitude as well of course of cloud cover, very different in Essex from that in Texas. In a study of 2000 horses, we examined 104 quarter horses in Essex UK and 102 from Texas USA, finding that the C_50_ for horses from Texas was 10.2 years while in those from Essex the value was 20.2 years, these statistically significantly different at *p* = 0.001. The proportion of Quarter horses at all ages with cataract was 0.24 in Essex and 0.58 in Texas, giving a relative risk ratio of 2.4. Diets and husbandry of quarter horses in Texas and Essex were not significantly different, involving grazing on pasture between 3 and 5 hours daily with additional hay and a limited addition of similar commercial concentrate diet given in stable. Given that Brentwood, Essex is located at a latitude of 51° North and Dallas Texas at 32° North, total hours of sunlight annually were estimated at 127 for Brentwood Essex and 237 for Dallas Texas with the strength of incident light due to differences in latitude being 54% of incident light and power at the equator for Essex and 84% in Texas. Percentage cloud cover over the two locations is more difficult to compare from metrological data given the relative rarity of dense cloud cover in Texas compared to Essex. The mean percentage of cloud cover is around 75% for Essex and 15% for Texas. Even given these difficulties in comparing meteorological data between the two locations, a conservative estimate for the increased solar irradiation in Texas as compared to Essex would suggest that the former has at least 3 times the ultraviolet irradiation of the later throughout the year, accounting for the significant difference in cataract prevalence between the two groups of animals. We also evaluated cataract prevalence in horses from Ghana accessed from military stables, private livery yards and racehorse stables finding a C_50_ value of 17.2 ± 4.2 years, lower than the value for horses in the UK which overall was 16.5 ± 3.6 years but here the influence of sunlight was more difficult to evaluate given that the management of the animals was very different from animals in the UK from a nutritional perspective as well as the time the horses were outside exposed to sunlight.

Increased ultraviolet irradiation is a particular problem nearer the south pole given the ozone hole or what might better be termed thinning of the ozone column over the Antarctic [14] with this leading to significant increased risk of pathologies such as skin cancer and cataract [15]. A study comparing cataract in dogs in New Zealand and the UK [16] showed a C_50_ value of 7.5 ± 3.9 for animals in New Zealand compared with 9.4 ± 3.3 years in the UK, these significantly different at *p *= 0.0047. All dogs in the New Zealand population studied had lens opacities by the age of 11.5 years compared with 13.5 years in the UK population. While diet and intake of drugs such as non-steroidal anti-inflammatories could not be compared between the two groups, this substantial difference in age-related cataract is highly likely to be caused by increased ultraviolet irradiation.

## Diet

One of the problems with determining the effect of diet on cataractogenesis in people is the difficulty of determining with any accuracy exactly what people’s dietary intake is. Also the duration of a study to show a significant change is long in human patients, with studies such as the VeCAT evaluation of the effect of vitamin E failing to see an effect after four years [17]. The Roche European American Cataract Trial (REACT) study, a randomized clinical trial to investigate the efficacy of an oral antioxidant micronutrient mixture to slow progression of age-related cataract showed a small but statistically significant effect after two years in the American but not the UK arm of the study [18]. One might think such a study would be easier with companion dogs since feeding a specific commercial companion animal food day in day out should allow a more specific estimation of nutritional intake and because cataract formation occurs over a much shorter time period in dogs which live for maybe 15 years and in whom metabolic and degenerative processes occur much more rapidly. Nevertheless, ensuring that people don’t feed their dogs treats and normalising other factors such as daylight exposure in companion dogs is complicated and examining companion animals regularly can be taxing. These issues have been ameliorated by examining a group of dogs kept in the same environment with specific diets to study nutritional influences on cognitive decline. Such an opportunity is provided by the CanCog beagle colony in Ontario, Canada. These animals are kept to investigate dietary influences on cognitive aging in the dogs.

We examined 144 dogs initially deemed cataract free by direct ophthalmoscopy and slit lamp biomicroscopy scoring them for presence of lens opacity annually over five years. The dogs were randomly assigned to groups supplemented with the plant-derived antioxidants alpha tocopherol and ascorbic acid, the mitochondrially-directed antioxidant L carnitine or alpha lipoic acid together with a control group of dogs fed a control diet without antioxidant supplementation. All supplements had an effect on cataract prevalence, yet it was only those dogs supplemented with alpha lipoic acid which demonstrated a statistically significant reduction in degree of lens opacification when compared with the control non-supplemented animals. Alpha lipoic acid is a potent antioxidant [19] commercially available in the canine dietary supplement Ocu-GLO™. Another study on nutritional influences evaluated 112 dogs with incipient cataracts and 60 dogs with immature cataracts yearly for 4 years [20, 21]. Both Ocu-GLO™ and Meni-One™, containing astaxanthin and curcuminoid, significantly delayed the progression of immature cataracts compared to the control group. The value of using dogs in such a study is the rapid development of lens opacities in middle aged dogs over the latter 4-5 years of their lives compared with the lengthy period required to evaluate such changes in human patients.

## Diabetes

Diabetes was an important D in Taylor’s original list of factors in cataractogenesis and this is particularly the case in companion animals. Across the animal kingdom diabetic hyperglycaemia has quite different effects on the lens. The rat rapidly develops diabetic cataract [22, 23] with the streptozotocin-induced diabetic rat being a major model for cataract formation [24]. The mouse appears, while not completely immune to such lens opacification, much less severely affected (Hegde et al 2003). The degu, a south American rodent, has the dual misfortune of developing diabetes frequently when fed sugar-rich diets unlike the coarse grass of its homeland but also in being exceptionally prone to diabetic cataract [25, 26]. These interspecific differences relate to the concentration of aldose reductase in the lens. This enzyme converts glucose to its sugar alcohol, sorbitol, but as the enzyme’s Km for sorbitol is much higher than that of hexokinase, at normal sugar concentrations in the lens glucose is converted to carbon dioxide and water with hexokinase enzymatically aiding the first step in that reaction chain. At higher intralenticular glucose concentrations only seen in diabetes mellitus, and once hexokinase is fully saturated, aldose reductase can convert the remaining glucose to sorbitol. This has a profound osmotic effect and draws water into the lens giving a cataract [27]. Inhibition of this enzyme can reduce or obviate diabetic cataractogenesis in experimental model animals [28–30] and possibly also in human patients [31]. Diabetic dogs develop mature lens opacities rapidly when intralenticular glucose levels are elevated in a manner not seen in human patients with a much lower activity of aldose reductase in the lens and so canine patients might act as a good model for investigating the inhibition of aldose reductase in slowing or preventing diabetic cataractogenesis.

In a study using alpha lipoic acid which, as well as being an antioxidant is an aldose reductase inhibitor, we took 30 dogs recently diagnosed with diabetes mellitus but without lens opacification and randomly assigned them either to an OcuGLO™ treatment group or a placebo group, the observer and dog owner masked to the nature of the treatment. Ten of the 15 dogs on the placebo developed significant cataract while on the study while 5 of the 15 on the OcuGLO™ developed lens opacification. Three of these 5 had concurrent pancreatitis and were regularly sick so may not have ingested the supplement. The mean time without change in lens opacification at the time of study completion was 201 ± 121 days with OcuGLO™ and 68 ± 28 days in the placebo group, this difference being statistically significant at *p *= 0.0003 [32]. A similar study using alpha lipoic acid alone gave equivalent results [33]. Whether supplementation would reduce the incidence of diabetic lens changes in human patients is currently unclear but would be well worth evaluating.

## Drugs

A key drug recognised to cause cataract in human patients is corticosteroid, these tending to manifest as posterior polar subcapsular opacities, described as granular opacities at the polar region of the posterior cortex, located just within the capsule [34]. It has to be said that such cataracts may be missed by general practitioner vets and dogs are unlikely to be noted to have visual problems which such minor opacities as they neither drive nor read much! Over a period of 9 months, 21 dogs receiving regular corticosteroid treatment for at least 90 days were identified and examined. Of those on oral steroid 19 of 21 had posterior polar subcapsular cataract while only 2 of the control 21 dogs had such opacities this significant at *p *< 0.05. Total dose was not significantly correlated to cataract prevalence and neither was duration of treatment but dose in mg/kg was significant at *p *= 0.046 and dose rate (mg/kg/day) significant at *p *= 0.016.

Experimental models for steroid induced cataract are few and far between. Laboratory rodents appear only rarely to develop the posterior polar subcapsular opacities seen in human steroid-induced cataracts [35]. It may be that the short lifespan of rats and rabbits does not allow significant protein change to show as a defined change in lens clarity. The fact that many dogs kept as companion animals are treated with steroid for a substantial period could make canine patients a valuable naturally occurred model for future research.

## Dehydration

Harding has particularly championed this as a major influence on the high prevalence of cataract in the tropical third world [36], suggesting that increased lens protein carbamylation may be responsible for the cataract formation with dehydration [37]. Other groups have not considered dehydration as important: those promoting daylight as the key factor in cataractogenesis in tropical areas may fail even to recognise it - Robman does not mention it in his survey of cataractogenesis from an Australian perspective where clearly dehydration is of little importance [38]. Consider India however and the picture is very different; Zodpey and colleagues working on a hospital-based population in Nagpur, Maharashtra State, show a relative risk of developing cataract of 3.1 in dehydrated individuals [39] while Minassian and co-workers, working on a similar central Indian population, estimated that dehydration from diarrhoea or heatstroke might account for around a third of cataract cases in the affected area [40]. A second study by this group in a different group both geographically and from a socioeconomic perspective strongly confirmed these findings with an estimated 38% of blinding cataract cases arising from repeated dehydrational crises [41] It was in view of this research that we sought to investigate cataract prevalence in dehydrated cats.

In our study comparing cataract prevalence in 2000 normal cats and 100 with a history of dehydrational crises, the C_50_ for cataract in dehydrated cats was 9.9 ± 2.5 years, differing from that in the normal population at 12.7 ± 3.4 years with a p value nearing significance (*p *= 0.06) [42]. All of the dehydrated cats had posterior cortical linear opacities similar to those seen in normal cats at a much greater age, although on occasion these were multiple and significantly more prevalent than those seen as a nature correlate of ageing. As with the steroid-treated dogs these cats may be a valuable spontaneously occurring population for further research.

## DNA

**Fig. 4:**
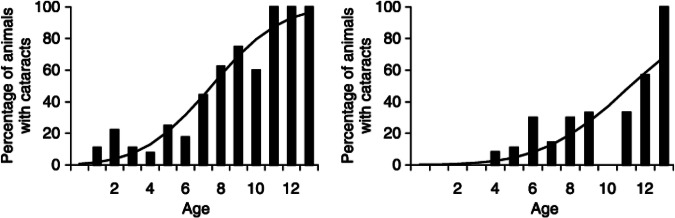
Prevalence curves for German Shepherd dogs (left) and West Highland White terriers (right) showing earlier onset of cataract in the German Shepherd dogs with a median longevity of 10.3 years comparted with the West Highland White Terrier with a median longevity of 12.8 years.

The genetic influence on age-related cataracts was not included by Taylor in his original list of Ds. Maybe we can add DNA into the group of Ds considered important factors in lens ageing? While clear genetic influence is seen in congenital cataracts, it can also play a key part in lens opacification in lens ageing too [43] Our study on 2000 dogs [10] showed marked difference in C_50_ between different breeds of dog. As an example, the German Shepherd Dog, with a median longevity of 10.3 years, C_50_ was 7.5 ± 3.1 years, while for the West Highland White Terrier, with a median longevity of 12.8 years, C_50_ was 11.2 ± 3.7 years. This interbreed difference, shown graphically in Fig. 4, was significant at *p *= 0.009. On one hand this seems obvious – a breed with an average longevity of say 12 years will naturally, one would anticipate, undergo cataract formation at a later age than one with an average lifespan of 10 years. The German Shepherd dog develops age-related cataract several years before the longer living West Highland White terrier. But what molecular changes are there between the lenses in these two breeds? Is there a difference in the ability of the chaperone molecule alpha crystallin to retard protein damage in the breed with greater longevity? Or are there changes in transcription of senescence genes in lens epithelial cells as has been shown in analysis of the transcriptome in ageing mouse lens? [44]. Mouse strains with different levels of oxidative-stress related damage have varying lifespans [45] and different rates of progression of age-related lens opacification too [46] although these do not seem to have been directly correlated in one coordinated study. Sadly, we have not, as yet, been able to answer these questions at a molecular level in any companion animal species.

Even so, evaluating cataract development in ageing individuals across different species can give valuable insights into ways of slowing cataractogenesis and potentially increasing lifespan as well [47], acting as a valuable stepping stone between laboratory rodent studies and human patients.

## Damage

A final D to be considered in cataract formation is damage [48]. A particular example of this involved scrub-jays kept in a facility where they were used for research on comparative cognition [49]. The birds were caught up in a net in their wire netting enclosure and over time developed cataracts. When a less invasive capture method was introduced cataract incidence dropped markedly, strongly suggesting that it was trauma of the birds colliding with their wire enclosure during capture which was leading to the cataract. A sizeable study showed one third of the 17,000 cases of ocular trauma in the United States Eye Injury Registry had lens opacification [50]. These patients had predominantly suffered single impact injuries, often catastrophic, but the birds in our study had multiple far less substantial traumas. A human equivalent might be lens injury in boxers. A study of 74 boxers nearly 40 years ago found 19% to have cataracts, 70% of them posterior polar subcapsular [51]. A more recent study of 35 boxers from Cameroon found 12% to have cataracts, some of them catastrophic in nature [52].

## Conclusion

It is hoped that this rapid canter through different causes of cataract in varying species has given a worthwhile insight into how spontaneously occurring lens opacities in companion animals can be a useful stepping stone from experimental models of lens opacification to methods of slowing development of cataract in human patients. Perhaps we cannot do anything about the genetic influences on human cataract formation and little about environmental impact on lens opacification, but maybe the dietary influences we have found to be valuable in slowing cataract formation in ageing or diabetic dogs might be worth evaluating in the human population.
